# Soluble and membrane-bound adenylate kinase and nucleotidases augment ATP-mediated inflammation in diabetic retinopathy eyes with vitreous hemorrhage

**DOI:** 10.1007/s00109-018-01734-0

**Published:** 2019-01-07

**Authors:** Julian Zeiner, Sirpa Loukovaara, Karolina Losenkova, Mariachiara Zuccarini, Ani M. Korhonen, Kaisa Lehti, Anu Kauppinen, Kai Kaarniranta, Christa E. Müller, Sirpa Jalkanen, Gennady G. Yegutkin

**Affiliations:** 1 MediCity Research Laboratory, University of Turku, Tykistökatu 6A, 20520 Turku, Finland; 2 Pharma Center Bonn, Pharmaceutical Institute, University of Bonn, Bonn, Germany; 3 Unit of Vitreoretinal Surgery, Department of Ophthalmology, Helsinki University Central Hospital and University of Helsinki, Helsinki, Finland; 4 Research Programs Unit, Genome-Scale Biology, University of Helsinki, Helsinki, Finland; 5 Department of Microbiology, Tumor and Cell Biology, Karolinska Institutet, Stockholm, Sweden; 6 School of Pharmacy, Faculty of Health Sciences, University of Eastern Finland, Kuopio, Finland; 7 Department of Ophthalmology, Kuopio University Hospital, Kuopio, Finland; 8 Department of Ophthalmology, Institute of Clinical Medicine, University of Eastern Finland, Kuopio, Finland; 9 Department of Molecular Genetics, Faculty of Biology and Environmental Protection, University of Lodz, Lodz, Poland

**Keywords:** Diabetic vitreous hemorrhage, Intravitreal ATP, Retina, Optic nerve head, Soluble adenylate kinase, Alkaline phosphatase, Ecto-nucleotidases

## Abstract

**Abstract:**

ATP and adenosine are important signaling molecules involved in vascular remodeling, retinal function, and neurovascular coupling in the eye. Current knowledge on enzymatic pathways governing the duration and magnitude of ocular purinergic signaling is incompletely understood. By employing sensitive analytical assays, this study dissected ocular purine homeostasis as a complex and coordinated network. Along with previously characterized ecto-5′-nucleotidase/CD73 and adenylate kinase activities, other enzymes have been identified in vitreous fluids, including nucleoside triphosphate diphosphohydrolase (NTPDase), adenosine deaminase, and alkaline phosphatase. Strikingly, activities of soluble adenylate kinase, adenosine deaminase, ecto-5′-nucleotidase/CD73, and alkaline phosphatase, as well as intravitreal concentrations of ATP and ADP, were concurrently upregulated in patients suffering from diabetic retinopathy (DR) with non-clearing vitreous hemorrhage (VH), when compared to DR eyes without VH and control eyes operated due to macular hole or pucker. Additional histochemical analysis revealed selective distribution of key ecto-nucleotidases (NTPDase1/CD39, NTPDase2, ecto-5′-nucleotidase/CD73, and alkaline phosphatase) in the human sensory neuroretina and optic nerve head, and also in pathological neofibrovascular tissues surgically excised from patients with advanced proliferative DR. Collectively, these data provide evidence for specific hemorrhage-related shifts in purine homeostasis in DR eyes from the generation of anti-inflammatory adenosine towards a pro-inflammatory and pro-angiogenic ATP-regenerating phenotype. In the future, identifying the exact mechanisms by which a broad spectrum of soluble and membrane-bound enzymes coordinately regulates ocular purine levels and the further translation of purine-converting enzymes as potential therapeutic targets in the treatment of proliferative DR and other vitreoretinal diseases will be an area of intense interest.

**Key messages:**

- NTPDase, alkaline phosphatase, and adenosine deaminase circulate in human vitreous.
- Purinergic enzymes are up-regulated in diabetic eyes with vitreous hemorrhage.
- Soluble adenylate kinase maintains high ATP levels in diabetic retinopathy eyes.
- Ecto-nucleotidases are co-expressed in the human retina and optic nerve head.
- Alkaline phosphatase is expressed on neovascular tissues excised from diabetic eyes.

**Electronic supplementary material:**

The online version of this article (10.1007/s00109-018-01734-0) contains supplementary material, which is available to authorized users.

## Introduction

Clear signaling roles for extracellular ATP, ADP, and adenosine have been established in virtually all organs and tissues [1], including the eye [2, 3]. ATP released from damaged neurons and activated microglia generally acts as a pro-inflammatory molecule initiating immunomodulatory, neurodegenerative, and hyperemic processes in the eye, which are mediated via activation of P2X7, P2Y_1_, and other ligand-gated (P2X) and G protein-coupled (P2Y) receptor subtypes co-expressed in the sensory retina and other ocular structures [2, 4–9]. Another mechanism of ATP action comprises its ectoenzymatic breakdown into adenosine, which in turn, mediates multiple effects (often counteracting to ATP) via binding to its own nucleoside-selective receptors. A_2A_ and other subtypes of adenosine receptors were shown to be implicated in the modulation of the light responses of photoreceptors, retinal hyperemia, pathological retinal angiogenesis, protection of neurons from hyper-excitation and glutamate toxicity [3, 10–13]. Previous research from our and other laboratories revealed the ability of VF and aqueous humor to maintain ATP and adenosine at certain characteristic nanomolar levels, which increase in pathological conditions, such as dry eye disease [14], glaucoma [2, 12, 15], age-related macular degeneration with subretinal hemorrhage [4], and diabetic retinopathy (DR) [16, 17].

DR is the most common microvascular complication of diabetes worldwide, characterized by visual impairment, vascular leakage, vessel occlusion, neuronal and glial dysfunctions, and cell death in retinal capillaries. The growth of newly formed abnormally differentiated vessels and non-clearing vitreous hemorrhage (VH) are clinical hallmarks in the proliferative form of DR (PDR) [18–23]. Treatments for the vision-threatening complications of DR have been greatly improved over the past decade. However, further development of preventional and interventional strategies against DR requires a better understanding of the exact mechanisms underlying diabetes-induced microvascular, neurodegenerative and inflammatory complications.

This study aims to further elaborate the role of purinergic mechanisms in the pathogenesis of DR. We have identified a broad spectrum of soluble and membrane-bound enzymes, which are co-expressed in the human VF, sensory retina and optic nerve head and coordinately regulate ocular nucleotide and nucleoside levels via two counteracting, purine-inactivating and ATP-regenerating, pathways. Furthermore, data on marked upregulations of intravitreal adenylate kinase (AK), ecto-5′-nucleotidase/CD73 (*e*N/CD73), adenosine deaminase (ADA), and alkaline phosphatase (ALP) activities and concurrently elevated ATP/adenosine ratio in DR eyes with VH provide a sufficient justification for reexamination of the role of purine homeostasis in the pathogenesis of DR.

## Materials and methods

### Surgical collection of vitreous samples and PDR neovascular tissues

A total of 136 DR patients and non-diabetic controls operated due to a quiescent idiopathic macular hole (MH) or pucker have been recruited to the original study (Table 1). Signed informed consent was obtained from each participant before insertion in the study. Clinical surgical enrollment criteria for diabetic vitrectomy were severe prolonged VH (> 2 months), traction retinal detachment or severe cystic macular edema with vitreous traction. VF samples were collected by a vitreoretinal surgeon at the start of the conventional three-port pars plana vitrectomy without an infusion of artificial fluid, as described elsewhere [24]. Additional information on vitreous collection and processing can be found in the Supplementary Material. For the preparation of serum, blood was drawn from the antecubital vein and allowed to clot before centrifugation (10 min at 1500*g*). Undiluted vitreous and serum aliquots were transferred into Eppendorf tubes and immediately frozen and stored at − 70 °C until laboratory analysis. Neovascular specimens were also excised surgically from vitrectomized eyes of two PDR patients during vitrectomy, using segmentation and delamination, cut with microscissors, and removed from the vitreous cavity with intraocular end-gripping microforceps (MaxGrip Alcon Laboratories), as described previously [22]. Extreme care was placed to remove intact, undamaged neovessels without causing damage to the optic nerve or temporal vascular arcade. The studies were conducted according to the principles of the Declaration of Helsinki and approved by the Institutional Review Board of the Helsinki University Central Hospital and the Ethical Committee.

**Table 1:** Diabetes mellitus, age and gender distribution among the patients studied

Patients	MH/pucker	PDR	NPDR
*n*	56	43	37
Males/females	18/38	25/18	24/13
DM1 / DM2	–	28/15	8/29
Age (years)	67.6 ± 0.8	45.3 ± 2.4	64.0 ± 2.3
Duration of DM (years)	–	24.0 ± 2.0 (43)	15.5 ± 2.0 (35)
VH/NH	0 / 56	34/9	14/23
Blood HbA1c (*%*)	–	9.3 ± 0.3 (34)	7.6 ± 0.4 (32)

The patients were sub-grouped as follows: non-diabetic eyes operated due to nonvascular vitreoretinal diseases, including quiescent idiopathic macular hole (MH) or pucker, as well as the diabetic group comprising of patients with proliferative (PDR) and non-proliferative (NPDR) forms of diabetic retinopathy. Prolonged vitreous hemorrhage (>2 months) has been used as an additional surgical enrolment criteria for distinguishing between the eyes without (NH) and with (VH) non-clearing hemorrhage. The levels of glycated hemoglobin A1c (*HbA1c*) in the whole blood and data on diabetes mellitus (DM) duration were available for the indicated patient number, as specified in the parentheses. Data are presented as mean ± SEM

### Preparation of human eye and PDR neovascular tissue sections

Cadaver eyeballs were enucleated 48 h postmortem from a female donor without apparent eye diseases. The use of human tissues and organs has been approved by the Ethical Committee of Turku University Hospital. Transverse sections of the retrobulbar optic nerve close to the optic nerve head, as well as neovascular tissues (excised surgically from PDR patients as described above), were embedded in the cryo-mold with Tissue-Tek® O.C.T. compound (Sakura Finetek Europe B.V. The Netherlands), cut using a cryostat and stored at − 80 °C. Eyeballs were additionally enucleated from a 68-year-old patient having a periocular tumor which was estimated to be invading to the bulbus. Immediately after the enucleation, the eyeballs were fixed in 10% formalin for 2–3 days followed by embedding in paraffin according to a routine protocol. Experienced pathologists confirmed that the tumor invasion had not reached the eyeball. The tenets of the Declaration of Helsinki and Kuopio University Hospital ethical rules were followed.

### Measurement of soluble purine-converting activities

Soluble NTPDase/ADPase, *e*N/CD73, ADA, and AK were assayed by incubating the VF (or serum) with [^3^H]ADP, [^3^H]AMP, [^3^H]adenosine, and [^3^H]AMP plus ATP, as respective enzyme substrates. ^3^H-labeled nucleotides and nucleosides were separated by thin-layer chromatography (TLC) and quantified by scintillation β-counting [17, 25] or developed by autoradiography, as described in the Supplementary Material. In competitive assays, the samples were pretreated for 30 min with inhibitors of NTPDases sodium polyoxotungstate-1 (POM-1) (Tocris Bioscience, Bristol, UK) and POM-144 [26], selective *e*N/CD73 inhibitors α,β-methylene ADP (APCP, Sigma) and its derivative N^6^-phenylethyl-[(phosphonomethyl)-phosphonic acid], (PSB-12379 [27]), inhibitor of AK diadenosine pentaphosphate (Ap_5_A), and ADA inhibitor erythro-9-(2-hydroxy-3-nonyl)adenine hydrochloride (EHNA, Tocris Bioscience). Soluble ALP was assayed by incubating VF (8 μl) on clear 96-well plates in 180 μl of basal salt solution (BSS; comprising 130 mM NaCl, 5 mM KCl, 1.5 mM CaCl_2_, 1 mM MgSO_4_, 25 mM HEPES and 5 mM glucose, pH 9.3) in the absence or presence of a specific ALP inhibitor tetramisole (5 mM). After a 60-min pre-treatment, 20 μl of chromogenic substrate for ALP, p-nitrophenyl phosphate (p-NPP), was added to the microwells at a final concentration of 3 mM, followed by incubation at 37 °C for 20 h and subsequent measurement of the absorbance intensity at 405 nm (Tecan Infinite M200, Salzburg, Austria). Likewise, serum ALP activity was determined after a 3-h incubation of human serum (2 μl) with 3 mM p-NPP.

### Determination of ATP, ADP, adenosine, and HbA1c

Intravitreal purine concentrations were determined by using bioluminescent (ATP and ADP) and fluorometric (adenosine) enzyme-coupled purine-sensing assays [28], under the conditions delineated experimentally for VF [16]. After defrosting, VF samples were immediately heat-inactivated for 5 min at 65 °C, thus preventing further metabolism of endogenous purines through soluble purinergic enzymes. The protein concentration was determined using the BCA Protein Assay Kit (Pierce, Rockford, IL). Glycated hemoglobin A1c (HbA1c) was measured in the blood using the Tina-quant® HbA1c assay kit with the Cobas Integra-800 analyzer (Roche Diagnostics, Rotkreuz, Switzerland).

### Immunohistochemical analysis of ecto-nucleotidases in the human eye

Formalin-fixed, paraffin-embedded sections of the human retina were stained for ecto-nucleotidases using UltraView DAB Detection Kit and BenchMark XT automated slide staining system (Ventana Medical Systems Inc., Roche, USA), according to manufacturer’s instructions. For immunofluorescence staining, cryosections of the optic nerve head and neovascular tissues were sequentially incubated with primary and secondary fluorescently labeled antibodies. Additional details are given in the Supplementary Material.

### Enzyme histochemistry

For localization of ecto-nucleotidase activities, a modification of the lead nitrate method was employed [29, 30], as described in the Supplementary Material. ALP activity was additionally evaluated by measuring dark purple precipitate after incubating the tissues at alkaline pH with artificial chromogenic enzyme substrates 5-bromo-4-chloro-3-indolyl-phosphate/nitro blue tetrazolium (BCIP/NBT, 0.35 mM each) [30].

### Statistical analysis

Two-tailed paired Student’s *t* test was used for single comparisons and one-way ANOVA with Dunnett’s multiple comparison post-hoc tests for multiple comparisons. Nonparametric Spearman’s correlation coefficients were computed to investigate the inter-correlations of continuous variables. In the case of comparative studies, the researcher(s) analyzed the encoded vitreous samples provided by the surgeon in a masked way, without knowing the clinical status of the patients. The results were analyzed with Prism GraphPad 7 software (San Diego, CA) and presented either as column bars (mean ± SEM) or as box-and-whiskers plots, where the box extends from the 25th to the 75th percentile with a horizontal line at the median and with whiskers showing the range of the data. The statistical significance levels were denoted as *P < 0.05 and **P < 0.01.

## Results

### A broad network of soluble enzymes co-exists in human vitreous and regulates intravitreal purine levels via counteracting, purine-inactivating and ATP-regenerating, pathways

**Fig. 1:**
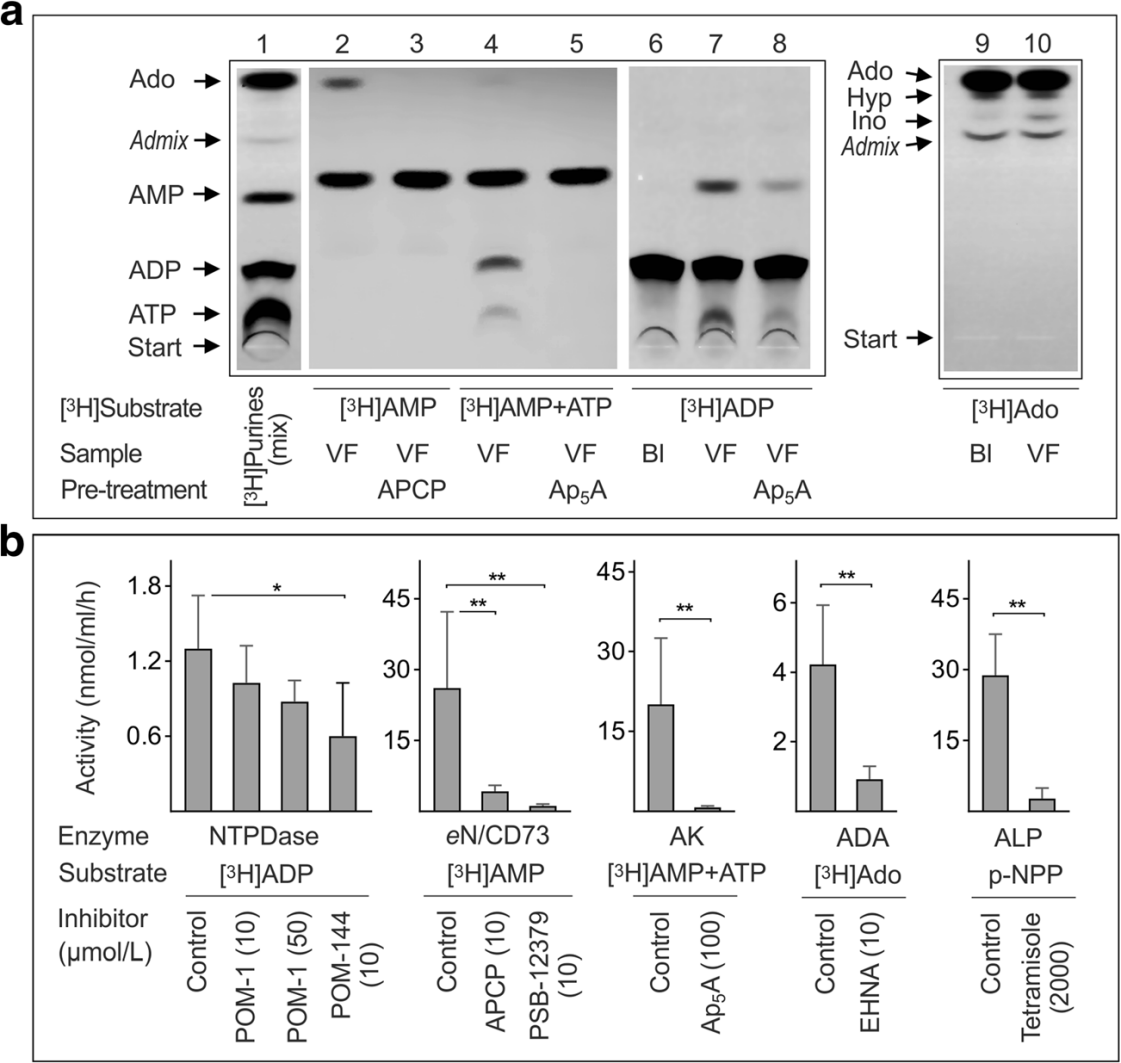
Competitive analysis of purine-converting pathways in human vitreous. **a** VF from macular hole or pucker (MH/Pucker) patients were pretreated with and without APCP and Ap_5_A followed by incubation with [^3^H]adenosine, [^3^H]ADP, [^3^H]AMP, and also [^3^H]AMP with ATP, as indicated. The blanks (Bl) show the radiochemical purity of tracer substrates in the absence of VF. Mixture aliquots were separated by TLC and developed by autoradiography. For pictorial illustration, stock solutions of [^3^H]ATP, [^3^H]ADP, [^3^H]AMP, and [^3^H]adenosine (~ 1 μl each) were pooled and separated by TLC (lane 1). The arrows indicate the positions of ^3^H-labeled nucleotides, adenosine (*Ado*), inosine (*Ino*), and hypoxanthine (*Hyp*), as well as the nonspecific admixture band (*Admix*) reflecting partial radiochemical impurity of the commercial [^3^H]adenosine preparation. **b** Vitreous purine-converting activities were quantified by TLC using [^3^H]ADP (18 μM), [^3^H]AMP (80 μM), [^3^H]AMP (200 μM) with 400 μM ATP and [^3^H]Ado (10 μM), as preferred substrates for NTPDase, eN/CD73, adenylate kinase (AK), and adenosine deaminase (ADA), respectively. Alkaline phosphatase (ALP) activity was also determined spectrophotometrically using an artificial enzyme substrate p-NPP (3 mM). The samples were pretreated without (Control) and with the indicated concentrations of certain enzyme inhibitors prior to the addition of substrates. The ordinates show specific activities expressed as nanomoles of substrates converted by 1 ml of fluid per hour (mean ± SEM, *n* = 4). *P < 0.05 and **P < 0.01, determined by Student’s *t* test (paired, two-tailed)

This study was undertaken to characterize the major purine-converting pathways in human vitreous. As shown in Fig. 1a, incubation of VF with [^3^H]AMP caused its breakdown into [^3^H]adenosine (lane 2), and this nucleotidase reaction was prevented with an eN/CD73 inhibitor, APCP (lane 3). In the presence of γ-phosphate-donating ATP, part of [^3^H]AMP was converted into high-energy ^3^H-phosphoryls (lane 4), whereas specific AK inhibitor Ap_5_A blocked this backward phosphotransfer reaction (lane 5). Incubation of VF with [^3^H]ADP as an initial substrate was accompanied by its transphosphorylation into [^3^H]ATP and [^3^H]AMP (lane 6), and this catalytic conversion was markedly inhibited in the presence of Ap_5_A (lane 7). Adding [^3^H]adenosine to the VF triggered its partial deamination into [^3^H]inosine (lane 10).

Consistent with the autoradiographic data, a direct quantitative analysis confirmed the presence of low but clearly detectable ADPase activity in most of the VF studied (Fig. 1b). Pre-treatment of the samples with a non-selective NTPDase inhibitor POM-144 [26], but not with another inhibitor POM-1, diminished the rate of [^3^H]ADP hydrolysis by ~ 50%. Taking into account the different ATP:ADP hydrolysis ratios for NTPDase1 and other members of this family, NTPDase2 and NTPDase3 (~ 1:1, 10–40:1 and 3–4:1, respectively) [31, 32] and by drawing an analogy with soluble NTPDase1/CD39 freely circulating in the human and murine blood [25], it may be speculated that NTPDase1/CD39 represents the major enzyme responsible for the measured [^3^H]ADP hydrolysis in human vitreous. The employment of [^3^H]AMP as another tracer substrate demonstrated the presence of AMPase activity, which can be blocked by *e*N/CD73 inhibitors, APCP and PSB-12397 (Fig. 1b). In the presence of γ-phosphate-donating ATP, part of [^3^H]AMP was phosphorylated into [^3^H]ADP/ATP, while Ap_5_A prevented this phosphotransfer reaction. For direct quantification of ADA activity, we used a particular solvent mixture that enabled better TLC separation of [^3^H]adenosine and its ^3^H-metabolites [33]. Incubation of VF with [^3^H]adenosine caused its deamination into [^3^H]inosine, which can be blocked by a specific ADA inhibitor EHNA (Fig. 1b). Soluble ALP was also determined spectrophotometrically. Due to the low sensitivity of this colorimetric technique, the assay settings have been modified and optimized accordingly, particularly by incubating the microplate for up to 20 h at alkaline pH and 37 °C. Long-term incubation of VF with p-NPP was accompanied by the development of a soluble yellow reaction product that may be detected at 405 nm, and this catalytic reaction was prevented in the presence of a specific ALP inhibitor, tetramisole (Fig. 1b). Collectively, the identification of a broad spectrum of purine-converting enzymes in human VF forms a solid background for further elucidation of the role of the ATP-adenosine axis in the pathogenesis of vitreoretinal diseases in humans.

### Soluble eN/CD73, ADA, AK, and ALP activities are selectively upregulated in diabetic retinopathy eyes with vitreous hemorrhage

**Fig. 2:**
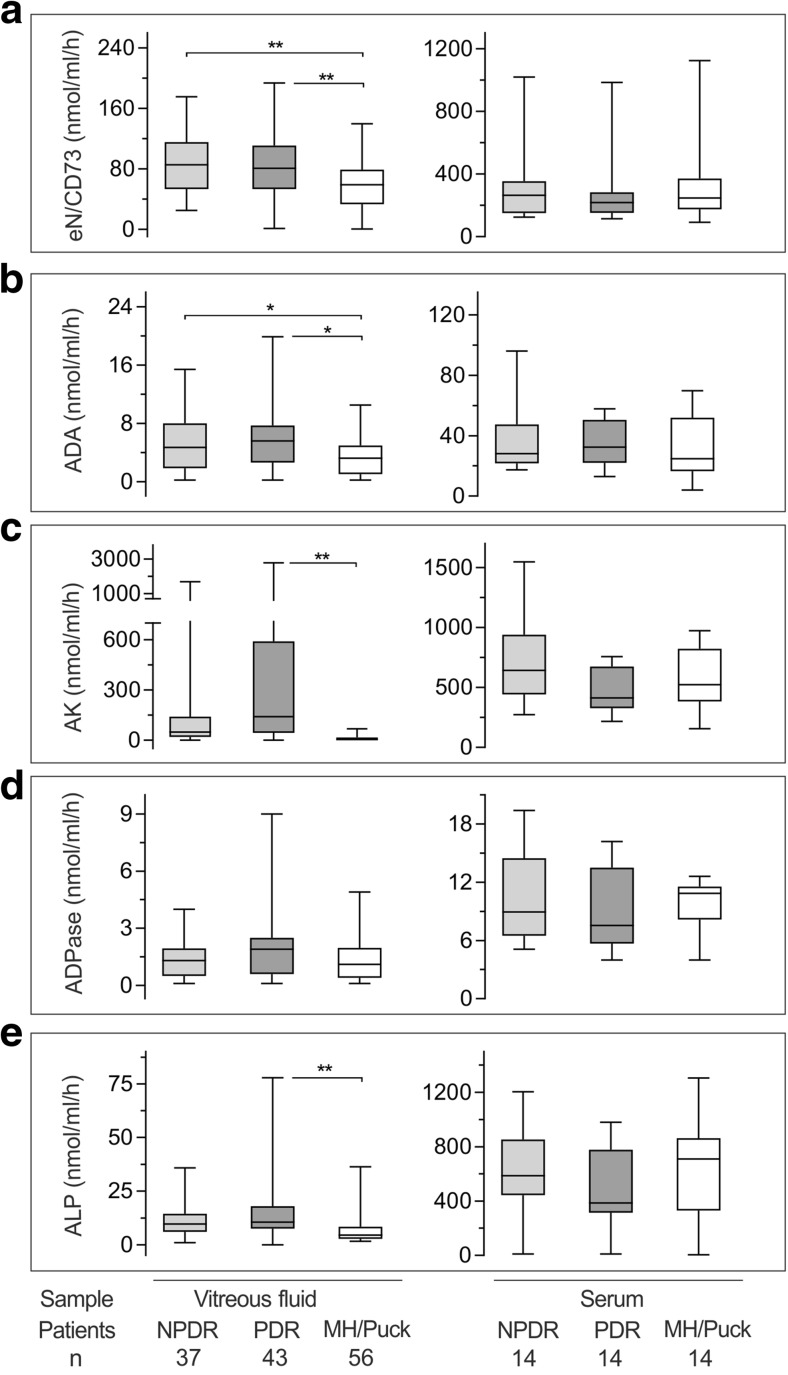
Analysis of soluble purine-converting activities in the vitreous fluids and sera from diabetic and non-diabetic patients. Specific ecto-5′-nucleotidase/CD73 (*e*N/CD73; **a**), adenosine deaminase (ADA; **b**), adenylate kinase (AK; **c**), NTPDase/ADPase (D), and alkaline phosphatase (ALP; **e**) activities were determined in the VF and serum samples collected from diabetic patients with non-proliferative (NPDR) and proliferative (PDR) forms of diabetic retinopathy, as well as control non-diabetic patients operated due to macular hole or pucker (MH/Puck). Purine-converting enzymes were assayed either by TLC with ^3^H-labeled purine substrates (**a**–**d**) or spectrophotometrically using the artificial ALP substrate p-NPP (**e**). Enzymatic activities are presented as box-and-whiskers plots expressed as nanomoles of substrates converted by 1 ml of fluid per hour. *P < 0.05 and **P < 0.01, determined by one-way ANOVA with Dunnett’s multiple comparison test

Next, we evaluated the pattern of intravitreal purine homeostasis at pathological states, particularly by comparing PDR and NPDR patients versus non-diabetic eyes operated due to MH or pucker. Detailed information on the type of diabetic mellitus, age and gender distribution among the patients studied is provided in Table 1. The ability of VF to metabolize ^3^H-labeled purines and p-NPP varied substantially among the individuals. Nevertheless, the activities of *e*N/CD73 (Fig. 2a), ADA (Fig. 2b) AK (Fig. 2c), and ALP (Fig. 2e), but not NTPDase/ADPase (Fig. 2d), were increased in VF from PDR patients, with the first two enzymes being also significantly upregulated in NPDR eyes. Additional comparative analysis of the corresponding soluble enzymatic activities in serum samples from diabetic and non-diabetic patients did not reveal any differences among the groups studied (Fig. 2a–e; right panels). These data suggest that the observed up-regulation of intravitreal enzymes occurs locally in DR eyes and is not accompanied by concurrent shifts in intravascular purine homeostasis in the systemic circulation. These findings are consistent with previous observations showing markedly increased intravitreal AK activities in PDR patients [17], and further demonstrated that other soluble enzymes (*e*N/CD73, ADA, and ALP) also became significantly upregulated in DR eyes.

**Fig. 3:**
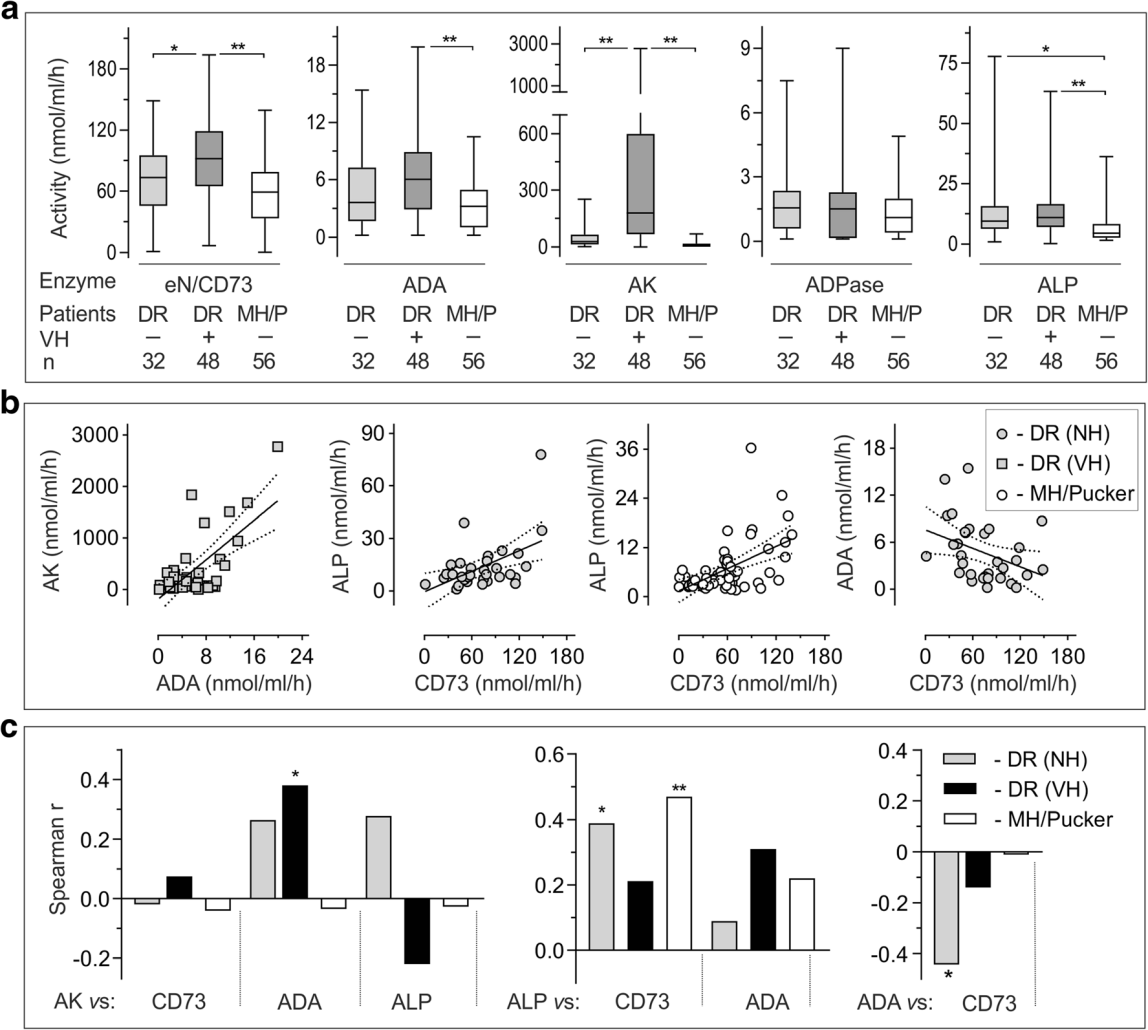
Soluble purine-converting activities are selectively up-regulated in diabetic eyes with vitreous hemorrhage. **a** Intravitreal *e*N/CD73, ADA, AK, NTPDase/ADPase, and ALP activities were determined in DR eyes without (−) and with (+) VH, as well as from non-diabetic eyes operated due to macular hole or pucker (MH/P). Enzymatic activities are expressed as nanomoles of substrates converted by 1 ml of fluid per hour and presented as box-and-whiskers plots. *P < 0.05 and **P < 0.01, determined by one-way ANOVA with Dunnett’s multiple comparison test. (**b**) Soluble purinergic activities were further correlated with each other. The most significant interrelations were observed by plotting ADA versus AK and eN/CD73, as well as ALP versus CD73 activities. The graphs display enzymatic activities determined in individual patients, as well as best-fit linear regression lines with 95% confidence intervals. Panel C summarizes the Spearman’s correlation coefficients (*Spearman r*) computed to investigate the relationships between the indicated enzymes measured both in diabetic and non-diabetic eyes. *P < 0.05 and **P < 0.01, determined by the nonparametric two-tailed correlation analysis

We also pooled the obtained AK data with similar activities determined previously under the same experimental settings in another cohort of patients operated due to DR (*n* = 86) and MH/pucker (*n* = 31) [17]. This combination allowed us to perform a more thorough and extended analysis of AK activities among the diabetic and non-diabetic groups. Surprisingly, intravitreal AK was shown to be selectively up-regulated only in DR eyes with severe prolonged VH, with no gender- or DM type-specific variations in the measured nucleotide-phosphorylating activities being observed within the subgroups studied (Supplementary Fig. S1). These data suggest that VH itself, rather than PDR, serves as a key determining factor associated with deregulated purine homeostasis in diabetic eyes. In fact, the performance of a new subgroup analysis based on this emerging criterion revealed high levels of eN/CD73, ADA, AK, and ALP in DR eyes with VH, which significantly exceeded respective soluble activities in DR eyes without VH and also in non-diabetic controls (Fig. 3a). Notably, all enzymatic activities were expressed as nanomoles of substrate(s) converted by 1 ml of fluid per hour. Additional spectrophotometric analysis did not reveal any differences in total intravitreal proteins among the groups studied, being equal to 5.10 ± 0.34 (mean ± SEM; *n* = 48), 4.94 ± 0.33 (*n* = 32) and 5.34 ± 0.16 (*n* = 56) mg/ml in the DR eyes with and without VH, as well as control MH/pucker eyes, respectively. These data allowed excluding the possibility that the revealed upregulation of soluble enzymatic activities might be due to the increased amounts of bulk proteins released into the vitreous cavity during VH, vascular leakage, diabetic tractional retinal detachment, or cell death.

### High intravitreal AK correlates with concurrently upregulated ADA activity and increased levels of ATP and ADP in DR eyes with VH

**Fig. 4:**
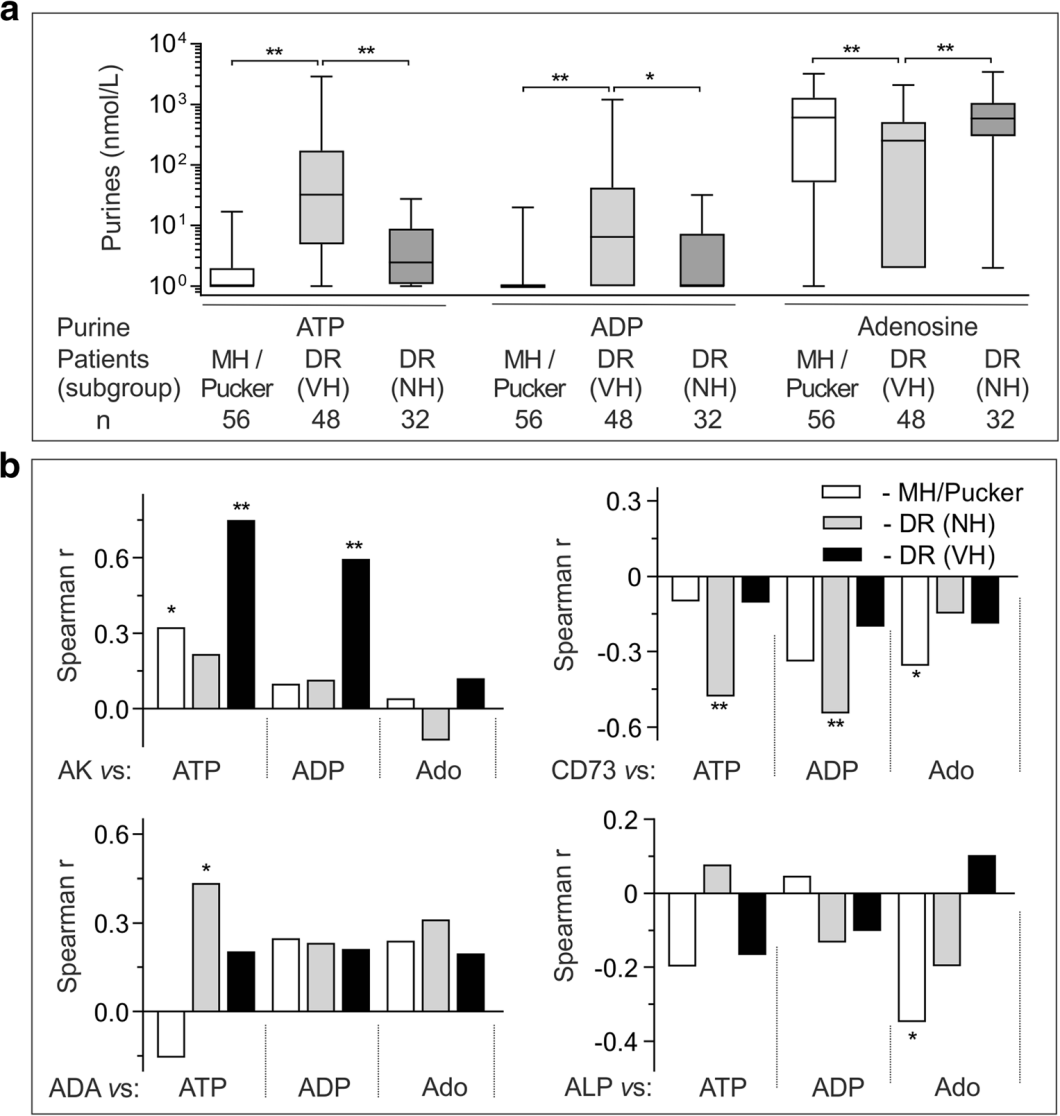
Soluble purinergic activities correlate with concurrently increased concentrations of ATP and ADP during diabetic vitreous hemorrhage. VF were collected from non-diabetic patients with macular hole or pucker (MH/pucker), as well as from DR patients, which were further sub-divided into groups of non-hemorrhagic eyes (NH) and eyes with VH. **a** Purine concentrations were assayed using enzyme-coupled bioluminescent (ATP, ADP) and fluorometric (adenosine) sensing assays and presented as box-and-whiskers plots (nmol/L). *P < 0.05 and **P < 0.01, determined by one-way ANOVA with Dunnett’s multiple comparison test. **b** Intravitreal purines were further correlated with soluble AK, *e*NCD73, ADA, and ALP activities, as indicated. Spearman’s correlation coefficients (*Spearman r*) were computed to investigate the relationship between soluble enzymatic activities and purine concentrations. Raw correlation analysis data for each particular enzyme are shown in Supplementary Figs. S3-S6. *P < 0.05 and **P < 0.01, determined by the nonparametric two-tailed correlation analysis

By nonparametric correlation analysis, we demonstrated the existence of a positive correlation between soluble AK and ADA activities in DR eyes with VH. eN/CD73 correlated with ALP both in diabetic and non-diabetic eyes and in addition, there was a significant inverse correlation between *e*N/CD73 and ADA activities in non-hemorrhagic DR eyes (Fig. 3b, c). Soluble purinergic activities were further correlated with ATP, ADP, and adenosine levels determined in the same vitrectomized eyes. Compared to diabetic eyes without VH and non-diabetic controls, DR eyes with VH were shown to maintain significantly increased ATP and ADP, as well as diminished adenosine concentrations (Fig. 4a and Supplementary Fig. 2S). Nonparametric correlation analysis demonstrated the existence of highly positive correlations between AK activity and intravitreal ATP and ADP levels in DR eyes with VH (Fig. 4b; Supplementary Fig. S3). Performance of a similar analysis for *e*N/CD73 revealed significant inverse correlations between this soluble nucleotidase and ATP and ADP levels in DR eyes without VH (Fig. 4b; Supplementary Fig. S4). The interrelations between other soluble enzymes, ADA, and ALP, and intravitreal purines were relatively modest or insignificant both in DR and non-diabetic groups (Fig. 4b; Supplementary Figs. S5 and S6).

### Immunohistochemical analysis reveals the selective distribution of key ecto-nucleotidases and ALP within the human sensory retina, PDR neovascular tissues, and optic nerve head

**Fig. 5:**
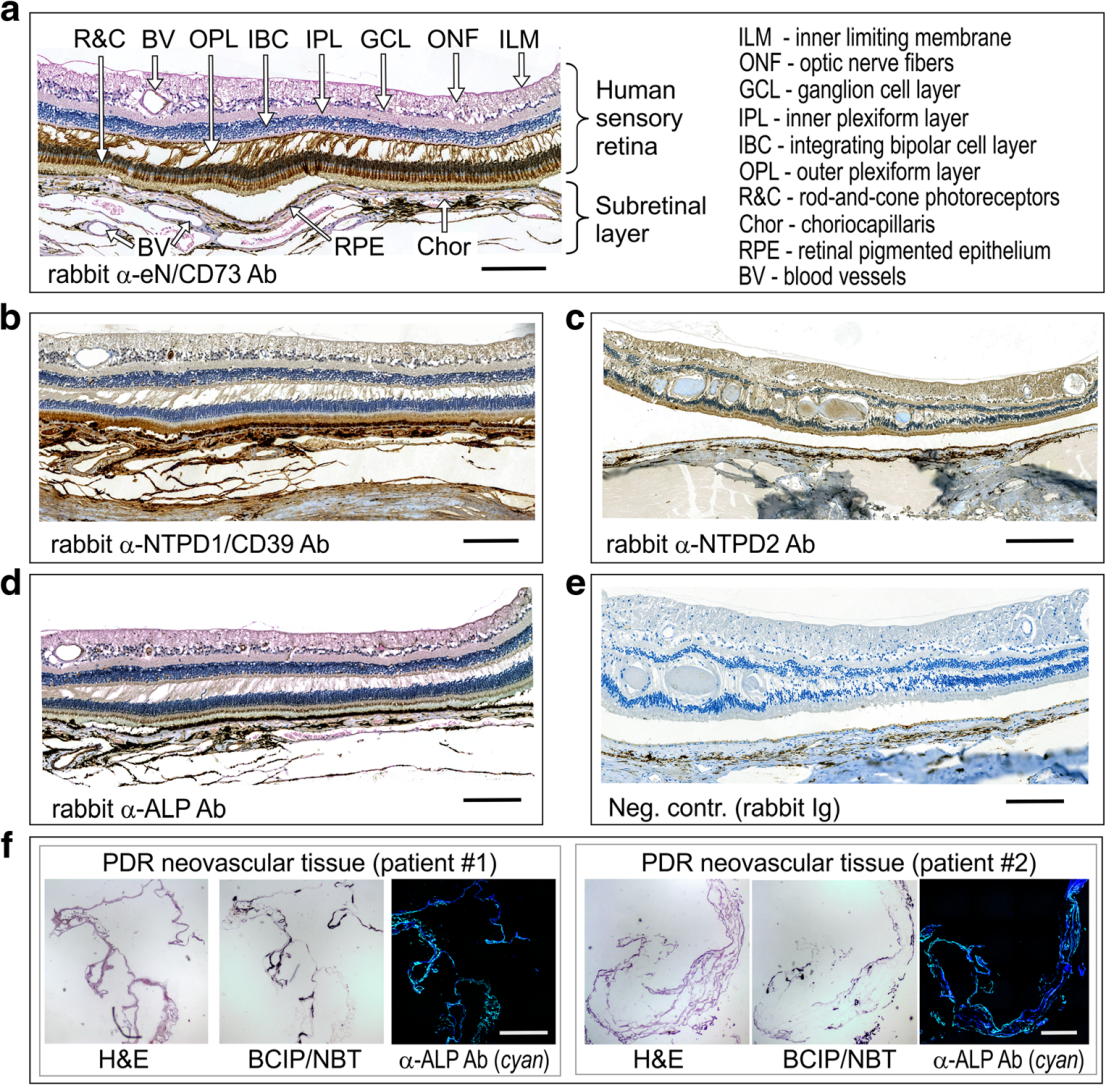
Distribution of ecto-nucleotidases and alkaline phosphatase in the human neuroretina and PDR fibrovascular tissues. Formalin-fixed, paraffin-embedded sections of an enucleated human eyeball were stained with rabbit anti-eN/CD73 (**a**), α-NTPDase1/CD39 (**b**), α-NTPDase2 (**c**), and α-ALP (**d**) antibodies, as well as with IgG from rabbit serum as a negative control (**e**). Subsequent microscopic examination reveals a selective distribution of key nucleotide-inactivating enzymes on photoreceptors, the choriocapillaris and other (sub)retinal structures. **f** Neofibrovascular tissues were surgically excised from two PDR patients and further assayed for ALP activity by using the artificial chromogenic enzyme substrates BCIP/NBT with subsequent monitoring of the development of the blue color reaction. Tissue cryosections were also co-stained with a rabbit anti-human ALP antibody (*cyan*) and DAPI (*blue*), as well as with hematoxylin and eosin (H&E), as indicated. Scale bars: 200 μm (**a**–**e**), 500 μm (**f**)

The current study was also aimed to characterize the distribution of major ecto-nucleotidases in the sensory retina and in this way, to establish a link between soluble and membrane-associated enzymes in the human eye. The presence of dark retinal pigmented epithelium (RPE) partially interferes with immunohistochemistry of the eye. Nevertheless, clear-cut differences in staining intensities were detected in the presence of antibodies against different nucleotidases (Fig. 5a–d), as compared with isotype-matched controls (Fig. 5e). In particular, *e*N/CD73 was shown to be expressed in the rod-and-cone-containing photoreceptor cells (Fig. 5a). The results also demonstrated selective compartmentalization of NTPDase1/CD39 (Fig. 5b), NTPDase2 (Fig. 5c), and ALP (Fig. 5d) in the neouroretinal and subretinal layers, including ganglion cells, blood vessels, choriocapillaris, and RPE.

Cryosections of the neovascular tissues surgically excised from two PDR eyes during vitrectomy, as well as the prelaminar region of the cadaver optic nerve head were also analyzed using enzyme (immuno)histochemistry. Measurement of ecto-nucleotidase and ALP activities, in combination with immunofluorescence staining, allowed us to pinpoint the distribution of key nucleotide-inactivating enzymes within the tissues. Incubation of neovascular tissues with BCIP/NBT (known as preferred chromogenic substrates for ALP [30]) revealed the development of dark blue precipitates in the connective tissues (Fig. 5f). Likewise, fairly similar staining patterns were observed by staining the tissues with an anti-ALP antibody (Fig. 5f; rightmost panels). Though, lead nitrate-based enzyme histochemistry did not detect any ecto-nucleotidase activities in the tissues studied (data not shown).

**Fig. 6:**
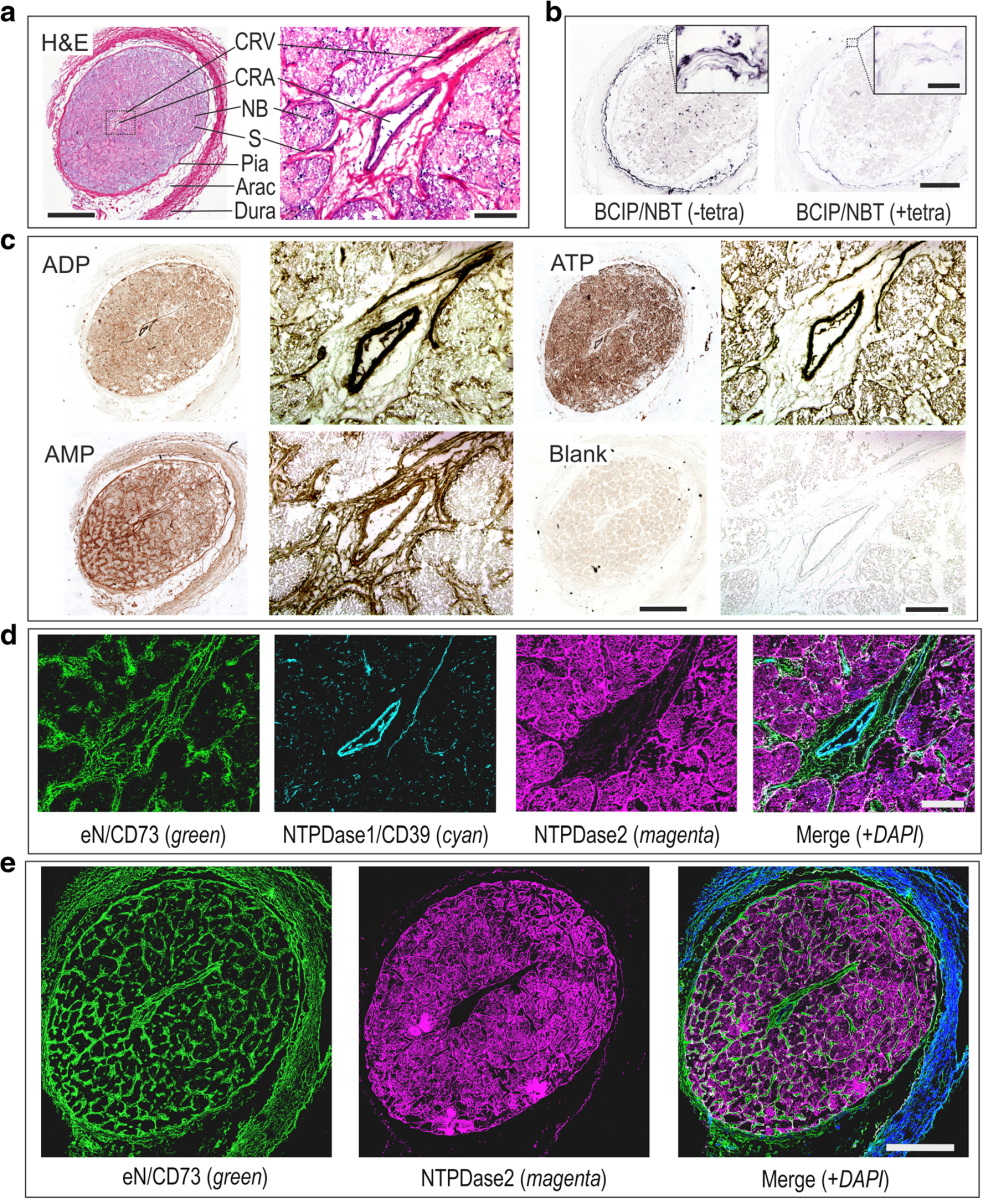
The distribution of ecto-nucleotidase activities in the human optic nerve head. **a** Hematoxylin and eosin (H&E) staining of transverse cryosections of the human optic nerve head. CRA, central retinal artery; CRV, central retinal vein; NB, nerve bundles; S, septa; Arac, arachnoid; Dura, dura mater. **b** Optic nerve cryosections were pre-incubated with or without specific ALP inhibitor tetramisole (tetra; 2 mM) and subsequently incubated with the mixture of artificial chromogenic substrates (BCIP/NBT, 0.35 mM each). A strong ALP-specific dark purple staining can be seen in the pia mater and connective tissue septa. (**c**) Lead nitrate-based histochemical staining of ecto-nucleotidase activities was performed in the absence (Blank) and presence of nucleotide substrates ADP (400 μM), ATP (400 μM) and AMP (2 mM), as indicated. (**d**, **e**) Tissue sections were incubated with mouse anti-CD73 (4G4), rabbit anti-NTPDase2 (hN2-2l), and guinea pig anti-NTPDase1/CD39 (hN1-1c) antibodies and subsequently co-stained with the appropriate secondary fluorochrome-conjugated immunoglobulins. Nuclei were counterstained with DAPI (shown in *blue* on the rightmost merged images). Scale bars: 1 mm (**a**, *left*; **b**, **c**, *left*), 200 μm (**a**, *right*; **c**, *right*, **d**, **e**), and 100 μm (**b**, *inset*)

Incubation of the optic nerve head with BCIP/NBT revealed the development of blue staining in the pia mater and blood vessels, which was prevented after pre-treating the sections with an ALP inhibitor tetramisole (Fig. 6b). Furthermore, we have demonstrated the presence of high ATP- and ADP-hydrolysing activities in the central retinal artery and other blood vessels (Fig. 6c), and further identified NTPDase1/CD39 as a major enzyme responsible for these nucleotidase reactions (Fig. 6d). The ADPase reaction product can also be seen, albeit faintly in comparison with ATP, in bundles of the optic nerve fibers (Fig. 6b), thus suggesting the presence of another ATP-inactivating enzyme, NTPDase2. Immunofluorescence staining confirmed that nerve bundle-surrounding microglial cells express NTPDase2 (Fig. 6e). Enzyme histochemistry assay with AMP (Fig. 6c), together with an *e*N/CD73 staining (Fig. 6d, e) revealed that the expression of *e*N/CD73 in the optic nerve was associated with the meningeal sheath and the connective tissue septa surrounding blood vessels and nerve bundles. These protein localization results allow the consideration of the innermost retina and optic nerve as potential sources of nucleotidases secreted to the vitreous cavity. Moreover, the pathological neofibrovascular tissue may be considered as yet another source of secreted ALP, contributing to the increased levels of this soluble enzyme in PDR eyes.

## Discussion

**Fig. 7:**
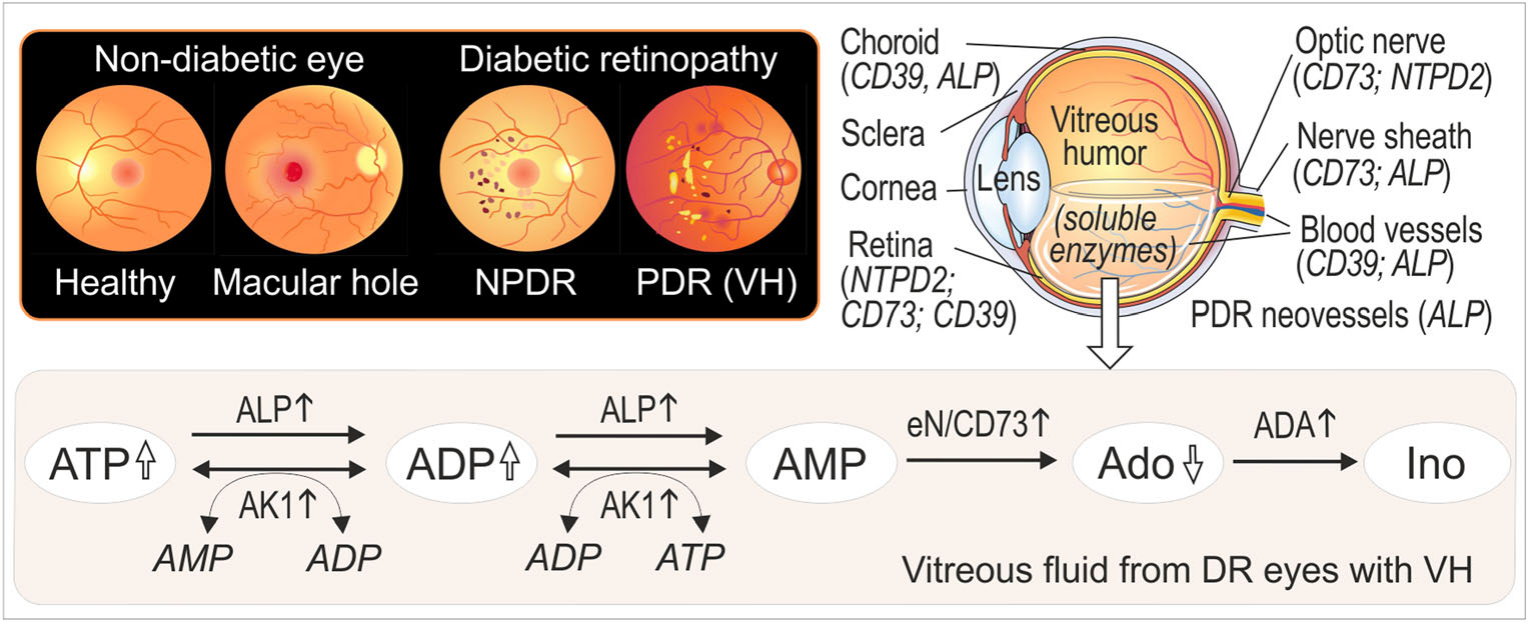
Ocular purine homeostasis is upregulated in DR eyes with VH. The cartoon illustrates the fundus of the eye from healthy subjects and patients with MH, as well as from non-proliferative diabetic retinopathy (NPDR) eyes and PDR eyes with prolonged non-clearing vitreous hemorrhage (VH). VH mainly occurs in PDR eyes, which are characterized by the invasion of abnormal blood vessels and neovascularization. The image on the right shows the cross section of the eye and further specifies the selective distribution of different ecto-nucleotidases in the sensory retina, blood vessels, and optic nerve head. The scheme highlights exchange activities of adenine nucleotides and adenosine in human vitreous. Soluble eN/CD73, ADA, AK, and ALP activities are up-regulated in diabetic eyes with VH, thus contributing to the persistent maintenance of high intravitreal concentrations of pro-inflammatory and angiogenic ATP

Purinergic signaling has become an area of renewed focus for both basic research and drug discovery. This study was undertaken to further elucidate regulatory mechanisms governing purinergic signaling in the human eye. Major findings are highlighted in Fig. 7 and can be summarized as follows: (1) a broad network of purine-converting enzymes was identified in human vitreous, where it coordinately controls purine levels through counteracting ATP-inactivating and ATP-regenerating pathways; (2) DR patients with VH contain high soluble AK, ADA, *e*N/CD73, and ALP activities, thus contributing to the maintenance of chronically elevated ATP/adenosine ratio; (3) this work additionally points to the selective distribution of key ecto-nucleotidases (NTPDase1/CD39, NTPDase2, *e*N/CD73, and ALP) within certain ocular structures, including neuroretinal and subretinal layers, the optic nerve head, and pathological PDR neofibrovascular tissues.

We have recently identified soluble intravitreal enzymes, *e*N/CD73 and AK (mainly comprising of the AK1 isoform) and further demonstrated that AK activity was selectively up-regulated in PDR eyes, accompanied by concurrently elevated concentrations of ATP, ADP, angiopoietin-2, TGFβ1, MMP-9, and other inflammatory and angiogenic factors [17]. The current study extends the earlier findings by showing that, along with *e*N/CD73 and AK, other soluble enzymes NTPDase/ADPase, ADA, and ALP circulate in the human vitreous. Strikingly, by dividing the VF based on non-clearing VH as additional clinical enrollment criteria for diabetic vitrectomy, we revealed highly specific upregulations of soluble AK, ADA, *e*N/CD73, and ALP activities in DR eyes with VH (see Fig. 3a).

Another salient finding of this study is the identification of an extensive network of ocular membrane-bound ectoenzymes. Previous histochemical analyses of human, canine and rodent eyes have shown a selective distribution of ecto-nucleotidases in the retinal vasculature (NTPDase1/CD39) and parenchyma (NTPDase2) [7, 34, 35], as well as inner Müller cell processes (*e*N/CD73) [7, 17, 34, 36]. Here, we have also demonstrated differential localizations of key ecto-nucleotidases in the human ganglion cells (NTPDase2), rod-and-cone-containing photoreceptor layer (*e*N/CD73), the outer segments of the photoreceptors, retinal vasculature and the adjacent RPE and choriocapillaris layers (NTPDase1/CD39) (see Fig. 5). Abundant expression of nucleotide-inactivating enzymes in the plane where the retina is cleaved in the living eye during pathological retinal detachment might be of particular relevance in terms of preventing excessive inflammatory responses and maintaining the choroidal and retinal blood supplies via rapid inactivation of locally released ATP. In this context, it is relevant to mention that P2X7 receptors expressed on photoreceptor cells can be implicated in ATP-mediated subretinal hemorrhage, choroidal neovascularization, and photoreceptor cell death [4, 7], while macrophage/microglial P2X7 seems to act as a scavenger receptor by clearing debris around the RPE and removing metabolic end products from photoreceptors [9].

Data on the specific distribution of ecto-nucleotidases in the optic nerve head further extend our knowledge on the regulatory mechanisms of purinergic signaling in the human eye. In particular, NTPDase1/CD39 was shown to be expressed in the central retinal artery and other ocular blood vessels. These findings are consistent with data on the abundant expression of NTPDase1/CD39 on vascular endothelial cells and its implication in the control of hemostasis through the termination of pro-inflammatory and pro-thrombotic effects of intravascular ATP and ADP [31, 32, 37]. By contrast, nerve bundle-surrounding microglial cells displayed strong immunoreactivity for NTPDase2. Due to the high preference of NTPDase2 for the hydrolysis of ATP over ADP [31, 32], this ectoenzyme presumably has functionality in the rapid scavenging of mitogenic extracellular nucleoside triphosphates in a neuronal environment, while the subsequent degradation of ATP-derived ADP into AMP will occur with considerable delay [38]. The identification of *e*N/CD73 in the connective tissue septa surrounding NTPDase2-positive microglia and NTPDase1/CD39-positive blood vessels suggest the efficient crosstalk between these ecto-nucleotidases, mediating tuned regulation of the ATP-adenosine axis within the optic nerve-fiber bundles and vasculature.

Tissue-nonspecific ALP is known to play a crucial role in bone and cartilage mineralization via the maintenance of the proper concentrations of a powerful mineralization inhibitor PP_i_ [32, 39] and it has also been identified among the top calcification-related genes overexpressed in the human trabecular meshwork [40]. Therefore, the selective expression of ALP in the neurosensory retina and optic nerve head (mainly restricted to the choriocapillaris, pia mater, and small blood vessels and capillaries) may be considered as an important and heretofore unrecognized mechanism controlling blood supply and soft tissue calcification. Furthermore, the presence of ALP in the pathological neofibrovascular tissues surgically excised from PDR eyes (see Fig. 5f), together with data on significantly upregulated soluble ALP activity in DR eyes without and with VH (see Fig. 3a) provide a novel insight into the role of this enzyme in the pathogenesis of advanced DR. Though, given a broad substrate specificity of ALP towards different phosphate-containing compounds with a pH optimum for this catalytic reaction lying in the alkaline range [31, 32], further studies are required to validate the contribution of ALP to the ocular nucleotide homeostasis.

Data on selective compartmentalization of ecto-nucleotidases in certain ocular structures, together with the identification of a wide spectrum of soluble enzymes co-expressed to a variable extent in human VF, provide novel insights into the regulatory mechanisms governing the duration and magnitude of purinergic signaling in a healthy and diseased eye. Taking into account the fact that the vitreous is the largest structure within the eye occupying about 80% of the ocular volume [41], the constitutive presence of soluble purine-converting enzymes might represent an important auxiliary effector system for tuned control of balanced nucleotides and adenosine levels in the VF. For instance, *e*N/CD73 is highly expressed on the photoreceptor cells in human (see Fig. 5a), canine [36, 42] and rodent [34] retinas, while A_2A_ receptors are known to be predominantly localized on the inner nuclear layer and ganglion cells facing towards the vitreous lumen [11, 36, 43]. These data indirectly imply an important physiological contribution of the soluble forms of *e*N/CD73 and ADA in controlling local adenosine levels at the vitreoretinal interface. The identification of soluble AK suggests that regulation of ocular nucleotide homeostasis extends beyond the inactivating pathways. AK is abundantly present in the cytosols of well-differentiated tissues with a high-energy demand [44, 45], including the brain [31, 46], and rod outer segments of the retina [47], where it regulates adenine nucleotide pools by catalyzing reversible phosphoryl transfers ATP + AMP ↔ 2ADP. Along with intracellular localization, this ATP-regenerating enzyme is expressed on the surface of vascular endothelial and other cells [31, 33] and it also freely circulates in the human and murine bloodstream [25, 48], human VF ([17]; current study), and rat pancreatic juice [49]. Strikingly, the dramatic activation of AK-mediated phosphotransfer reactions in DR eyes with VH correlates with concurrently elevated concentrations of ATP and ADP (see Fig. 4b), thus allowing to consider this soluble enzyme as an unfavorable factor triggering spatial propagation of ATP-mediated inflammatory responses into the surrounding retina far distant from the site of focal detachment.

These findings prompted important questions regarding the origins of the measured activities and mechanisms underlying their appearance in the vitreous. It may be speculated that certain portions of ocular (ecto)enzymes are co-released into the vitreous cavity, along with ATP and other transmitters, in response to light-evoked neuronal stimulations, mechanical perturbations caused by incessant eye movements and variations in the vascular tone and intraocular pressure. Cellular mechanisms underlying the release of purinergic enzymes might particularly involve the shedding of membrane-bound *e*N/CD73 and ALP via cleavage of their glycosyl-phosphatidylinositol anchors, microvesicular shedding, exocytosis, and other secretory pathways [31, 32]. Therefore, in principle, high soluble activities in the DR eyes might reflect a global upregulation of membrane-bound ectoenzymes and their partial shedding into the vitreous lumen. Indeed, the activities of ecto-nucleotidases and other components of the purinergic signaling cascade are known to be activated in the retina under all pathophysiological conditions investigated so far [4, 5, 7, 13]. For instance, the expression of A_2A_ receptors and the activity of *e*N/CD73 are upregulated in the inner Müller cell processes during the vasculoproliferative stage of an experimental model of oxygen-induced retinopathy triggered by a 4-day exposure of neonatal dogs to high oxygen and their subsequent return to normal air [36]. Though, our data on markedly activated intravitreal purine homeostasis only in the DR eyes with severe VH suggest the implication of more specific mechanisms triggering the release of soluble enzymes and/or endogenous ATP from the extravascular blood cells and abnormal blood vessels in the course of retinal degeneration, blood clotting, and neovascularization.

Collectively, these data provide evidence for selective VH-related shifts in purine homeostasis in DR eyes from the generation of anti-inflammatory adenosine towards a pro-inflammatory and angiogenic ATP-regenerating phenotype. Identifying the exact mechanisms by which the extensive network of membrane-associated and soluble enzymes regulates ocular nucleotide and nucleoside concentrations and the further translation of purine-converting enzymes as potential therapeutic targets in the treatment of proliferative retinopathies with VH and other vascular and degenerative eye diseases will be an area of intense interest in the future.

## Electronic supplementary material

ESM 1 (DOCX 1904 kb)
