# Supplementary material for: Soluble and membrane-bound adenylate kinase and nucleotidases augment ATP-mediated inflammation in diabetic retinopathy eyes with vitreous hemorrhage
Source: J Mol Med (Berl). 2019 Jan 7;97(3):341–54. doi: 10.1007/s00109-018-01734-0 (PMC6394560; doi:10.1007/s00109-018-01734-0)
Supplement: Supplementary file 1 — (DOCX 1904 kb) [file 109_2018_1734_MOESM1_ESM.docx]

**Supplementary data for the manuscript: “Soluble and Membrane-bound Adenylate Kinase and Nucleotidases Augment ATP-mediated Inflammation in Diabetic Retinopathy Eyes with Vitreous Haemorrhage”, by Julian Zeiner and co-authors**

**Supplementary Materials and Methods**

**Study subjects and sample collection**

VF samples from 136 patients recruited in the Unit of Vitreoretinal Diseases, Helsinki University Central Hospital were available for analysis. The study was conducted according to the principles of the Declaration of Helsinki, and it was approved by the Institutional Review Board of Helsinki University Central Hospital and the Ethical committee. A signed informed consent was obtained from each participant before insertion in the study. All patients underwent proper eye examination by the recruiting surgeon preoperatively, which included measurement of visual acuity, intraocular pressure, axial length and biomicroscopy of the anterior and posterior segment of the eye. The clinical surgical enrolment criteria for diabetic vitrectomy were prolonged non-clearing vitreous haemorrhage (VH) (>2 months), traction retinal detachment or severe cystic macular oedema with vitreomacular traction [[1](#_ENREF_1)]. Preoperative injections of VEGF inhibitors were not given to the enrolled diabetic study eyes, neither were any VEGF inhibitors used intraoperatively. Diabetic eyes were sub-divided into PDR and NPDR groups. In the eyes with PDR, perfused neovessels were observed on the retina or optic disc during vitrectomy. Non-diabetic patients operated because of idiopathic macular hole or quiescent pucker were recruited as controls. Detailed information on the type of diabetic mellitus, age and gender distribution among the patients studied is provided in Table 1. Additional analysis of the glycemic status confirmed that in comparison with a normal reference range of 4-6% determined in healthy persons (data not shown), the levels of glycated HbA1c were markedly elevated in the blood of diabetic patients (Table 1). All vitrectomies were performed by a vitreoretinal surgeon. Undiluted vitreous samples (up to 1000 µl) were collected at the start of the conventional 3 port pars plana vitrectomy (20G or 23Gauge, Accurus, Alcon Instruments, Inc., Fort Worth, TX, USA) without an infusion of artificial fluid. The samples were collected by manual aspiration into a syringe via the vitrectomy with the cutting function activated. For the preparation of serum, blood was drawn from the antecubital vein into silicone-containing tubes and allowed to clot before centrifugation (10 min at 1500g). Vitreous and serum sample aliquots were transferred into sterile 1.5 ml Eppendorf tubes (Freemont, CA, USA) and immediately frozen and stored at −70°C until laboratory analysis.

**Thin-layer chromatographic (TLC) analysis of purine-converting activities**

Soluble purine-converting activities were determined by thin-layer chromatography (TLC) using [2,8-^3^H]ATP and [2,8-^3^H]ADP (Perkin Elmer, Boston, MA), [2-^3^H]AMP (Quotient Bioresearch, GE Healthcare, Rushden, UK), and [2,8-^3^H]adenosine (Hartman Analytic, Braunschweig, Germany) as appropriate substrates. The standard assay contained, in a final volume of 80 μl of RPMI 1640 medium supplemented with 25 mM HEPES (pH 7.35), 4 mM β-glycerophosphate, 5 μl of VF (or 2 µl of serum) as the enzyme source, and different concentrations of unlabelled substrate(s) with tracer ^3^H-nucleotide or adenosine (~4×10^4^ dpm). Specifically, (i) for nucleoside triphosphate diphosphohydrolase (NTPDase) assay, the samples were pre-treated for 20 min with 80 μM diadenosine pentaphosphate (Ap_5_A, Sigma), followed by a 65-min incubation with 18 μM [2,8-^3^H]ADP (Perkin Elmer, Boston, MA); (ii) *e*N/CD73 activity was determined after a 60-min incubation of specimens with 80 µM [2-^3^H]AMP (Quotient Bioresearch, GE Healthcare, Rushden, UK); (iii) AK was assayed after a 45-min incubation with 200 μM [^3^H]AMP as a phosphorus acceptor in the presence of 400 μM γ-phosphate-donating ATP; (iv) ADA was measured after a 60-min incubation with 10 μM [2-^3^H]adenosine (Hartman Analytic, Braunschweig, Germany). Catalytic reactions were terminated by applying aliquots of the mixture onto Alugram SIL G/UV_254_ sheets (Macherey-Nagel, Duren, Germany). ^3^H-labeled nucleotides and nucleosides were separated by TLC using isobutanol/isoamyl alcohol/ 2-ethoxyethanol/ ammonia/H_2_O (9:6:18:9:15), visualized under UV light, scraped and quantified by scintillation β-counting. For the adenosine deaminase assay, we used a particular solvent mixture comprising of isobutanol/ethylacetate/methanol/ ammonia (7:4:3:4) that enabled better separation of [^3^H]adenosine and its ^3^H-metabolites, inosine and hypoxanthine [[2](#_ENREF_2)]. Enzymatic activities were expressed as nanomoles of ^3^H-substrates metabolized by milliliter of fluid per hour. TLC plates were also exposed to BioMax MS films (Carestream Inc, Rochester, NY) for one month at -70°C with the subsequent autoradiographic analysis.

**Immunohistochemical analysis**

Formalin-fixed, paraffin-embedded sections of the eye were stained for ecto-nucleotidases using UltraView DAB Detection Kit and the BenchMark XT automated slide staining system (Ventana Medical Systems Inc, Roche, USA), according to manufacturer’s instructions. The sections were deparaffinized, pre-treated with Cell Conditioning Solution (CC1) and sequentially incubated for 60 min at 37ºC with rabbit antibodies against *e*N/CD73 (D7F9A, Cell Signaling Technology), ALP (MBS9125423, MyBioSource Inc., San Diego, CA), NTPDase1/CD39 (hN1-9l) and NTPDase2 (hN2-2l) (provided by Prof. Jean Sevigny, Quebec, Canada; http.//ectonucleotidases-ab.com) at 1:100-1:200 dilutions, as well as with IgG from rabbit serum (1 µg/ml, 15006, Sigma) as a negative control. Stained slides were counterstained with Mayer’s hematoxylin, mounted with DPX Mountant (Sigma) and captured using a Pannoramic 250 slide scanner (3DHistech Ltd., Budapest, Hungary).

**Immunofluorescence staining**

**C**ryosections of the optic nerve head and neovascular tissues were thawed and incubated for 60 min at room temperature with the following primary antibodies (diluted in PBS at 1:100-500): guinea pig anti-NTPDase1/CD39 (hN1-1c), rabbit anti-NTPDase2 (hN2-2L) and pre-immune sera from the same animals (http.//ectonucleotidases-ab.com; [[3](#_ENREF_3), [4](#_ENREF_4)]) polyclonal rabbit anti-ALP antibody (MyBioSource Inc.), as well as mouse anti-CD73 mAb (4G4) and isotype-specific antibody against chicken T cells (3G6) [[4](#_ENREF_4), [5](#_ENREF_5)]. The sections were subsequently co-stained with secondary goat α-mouse Alexa Fluor^®^ 488- and goat α-rabbit Alexa Fluor^®^ 647-conjugated (Invitrogen Life Technologies) and Cy3-conjugated donkey anti-guinea pig (Jackson ImmunoResearch Laboratories Inc) immunoglobulins diluted in PBS at 1:500-1000. Extensive washes with PBS were performed after each step. The slides were mounted with ProLong Gold Antifade medium containing DAPI (Invitrogen). Multichannel fluorescence images were captured using a confocal spectral laser-scanning microscope Zeiss LSM780 (Zeiss GmbH, Oberkochen, Germany) using EC Plan-NEOFLUAR 10×/0.30 or Plan-APOCHROMAT 20×/0.8 objectives. Images were further edited using Fiji-Image J software (version v1.52h).

**Enzyme histochemistry**

For localization of nucleotidase activities, the lead nitrate method was employed [[6](#_ENREF_6), [7](#_ENREF_7)]. In brief, tissue cryosections were pre-incubated for 45 min in Trizma-maleate buffer (TMB; 40 mM Trizma® maleate, pH 7.4) containing 0.25M sucrose and the inhibitor of ALP, tetramisole (2 mM). The enzymatic reactions were performed at 37ºC in TMB supplemented with 0.25M sucrose, 0.5 mM CaCl_2_ , 2 mM tetramisole, 2 mM Pb(NO_3_)_2_ and one of the following phosphorylated compounds: ATP, ADP (preferred substrates for NTPDases; 400 μM each, 45-min incubation) and AMP (substrate for *e*N/CD73; 2 mM, 2-hour incubation). In blank specimens, the substrate was omitted from the incubation solution. The lead orthophosphate precipitated in the course of nucleotidase activity, and it was visualized as a brown deposit by incubating the sections for 30 seconds in 0.5% (NH_4_)_2_S. The activity of ALP was additionally visualized as dark blue precipitate after incubating the sections for 1 hour at 37ºC in 40 mM TMB (pH 9.3) containing 0.25M sucrose, 5 mM MgCl_2_, and mixture of artificial chromogenic substrates 5-bromo-4-chloro-3-indolyl phosphate/nitro blue tetrazolium (BCIP/NBT, 0.35 mM each) [[6](#_ENREF_6)]. Generation of the insoluble reaction product NBT diformazan was directly visualized by a progressively increased blue staining intensity. Slides were washed three times in TMB and mounted with Aquatex (Merck). Tissue sections were also stained with haematoxylin and eosin and mounted with DPX Mountant (Sigma). Bright-field microscopy images were captured using the Olympus BX60 microscope equipped with an Olympus DP71 camera using an UPlanFL 10×/0.30 objective (Olympus, Tokyo, Japan). Multiple images of adjacent areas were captured using Zeiss AxioVert 200M microscope with AxioCam MRc camera using A-Plan 10×/0.25 objective, and then combined via stitching to a larger overview using the MosaiX software module of AxioVision 4.8.2 software (Zeiss GmbH, Oberkochen, Germany). The images were captured at identical exposure times for all substrates employed, and also for blank samples, and further edited in parallel using Adobe Photoshop CS6 software.

**Supplementary references**

1. Loukovaara S, Robciuc A, Holopainen JM, Lehti K, Pessi T, Liinamaa J, Kukkonen KT, Jauhiainen M, Koli K, Keski-Oja J, et al. (2013) Ang-2 upregulation correlates with increased levels of MMP-9, VEGF, EPO and TGFbeta1 in diabetic eyes undergoing vitrectomy. Acta Ophthalmol 91: 531-539

2. Yegutkin GG, Samburski SS, Jalkanen S (2003) Soluble purine-converting enzymes circulate in human blood and regulate extracellular ATP level via counteracting pyrophosphatase and phosphotransfer reactions. Faseb J 17: 1328-1330

3. Liu X, Yu L, Wang Q, Pelletier J, Fausther M, Sevigny J, Malmstrom HS, Dirksen RT, Ren YF (2012) Expression of ecto-ATPase NTPDase2 in human dental pulp. J Dent Res 91: 261-267

4. Losenkova K, Zuccarini M, Helenius M, Jacquemet G, Gerasimovskaya E, Tallgren C, Jalkanen S, Yegutkin GG (2018) Endothelial cells cope with hypoxia-induced depletion of ATP via activation of cellular purine turnover and phosphotransfer networks. Biochim Biophys Acta 1864: 1804-1815

5. Airas L, Niemela J, Salmi M, Puurunen T, Smith DJ, Jalkanen S (1997) Differential regulation and function of CD73, a glycosyl-phosphatidylinositol-linked 70-kD adhesion molecule, on lymphocytes and endothelial cells. J Cell Biol 136: 421-431

6. Langer D, Hammer K, Koszalka P, Schrader J, Robson S, Zimmermann H (2008) Distribution of ectonucleotidases in the rodent brain revisited. Cell Tissue Res 334: 199-217

7. Mercier N, Kiviniemi TO, Saraste A, Miiluniemi M, Silvola J, Jalkanen S, Yegutkin GG (2012) Impaired ATP-Induced Coronary Blood Flow and Diminished Aortic NTPDase Activity Precede Lesion Formation in Apolipoprotein E-Deficient Mice. Am J Pathol 180: 419-428

8. Loukovaara S, Sandholm J, Aalto K, Liukkonen J, Jalkanen S, Yegutkin GG (2017) Deregulation of ocular nucleotide homeostasis in patients with diabetic retinopathy. J Mol Med (Berl) 95: 193-204

**
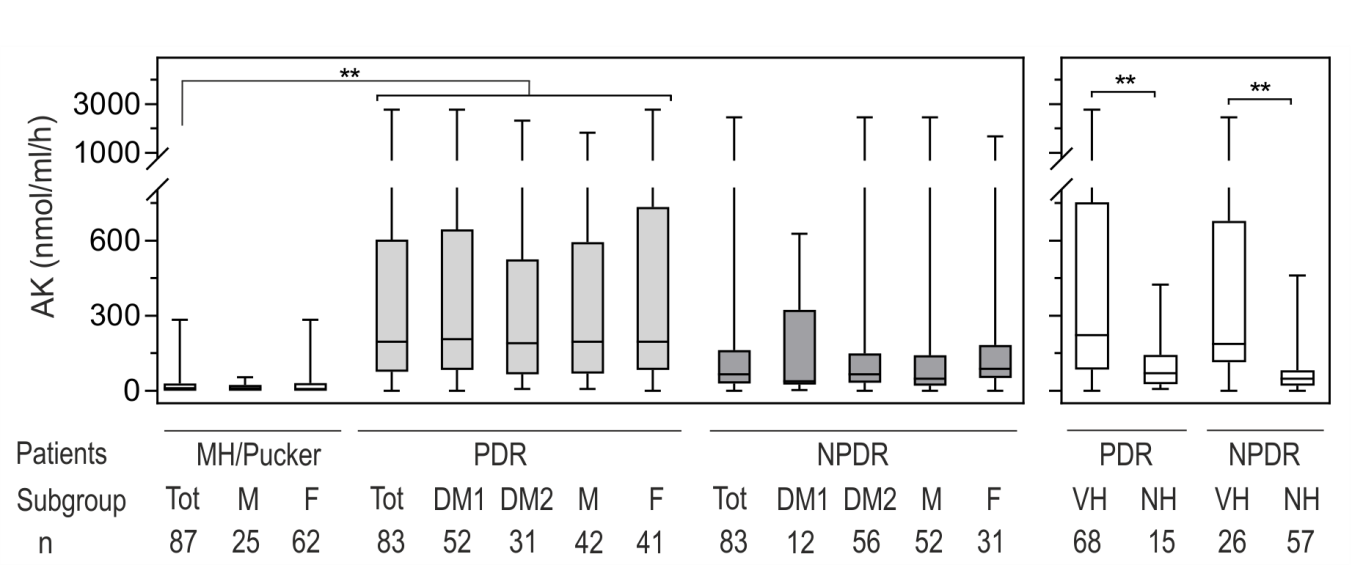
**

**Supplementary Fig. S1.** Soluble adenylate kinase is selectively up-regulated in DR eyes with vitreous haemorrhage. Vitreous samples from diabetic (n=80) and non-diabetic (n=56) eyes were assayed for AK, and the results obtained were pooled with similar enzymatic activities determined previously in another cohort of patients operated due to DR (n=86), MH and pucker (n=31) [[8](#_ENREF_8)]. This combined analysis allowed us to compare soluble AK activities among the different subgroups, including males (*M*) and females (*F*), diabetes mellitus-1 (*DM1*) and -2 (*DM2*), proliferative (*PDR*) and non-proliferative (*NPDR*) forms of diabetic retinopathy, as well as diabetic eyes without (*NH*) and with severe prolonged vitreous haemorrhage (*VH*). Enzymatic activities are expressed as nanomoles of [^3^H]AMP-ATP transphosphorylated by one milliliter of fluid per hour. **P<0.01, determined by one-way ANOVA with Dunnett’s multiple comparison test.


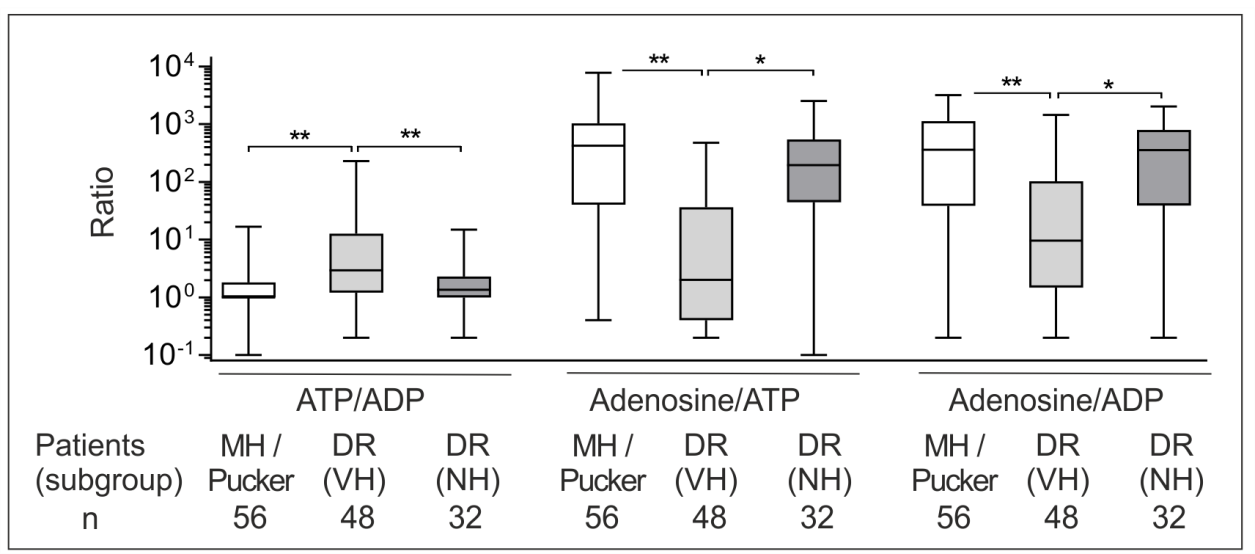


**Supplementary Fig. S2.** Intravitreal ATP and adenosine levels are inversely up- and down-regulated in diabetic eyes with vitreous haemorrhage. VF were collected from non-diabetic patients operated due to macular hole or pucker (MH/Pucker), as well as from DR patients, which were further sub-divided into groups of non-haemorrhagic eyes (NH) and eyes with VH. Intravitreal purine levels were determined using enzyme-coupled bioluminescent (ATP, ADP) and fluorometric (adenosine) sensing assays (*see Figure 4*). The graph shows the ratios between the measured purine concentrations presented as box-and-whiskers plots. *P<0.05 and **P<0.01, determined by one-way ANOVA with Dunnett’s multiple comparison test.

**
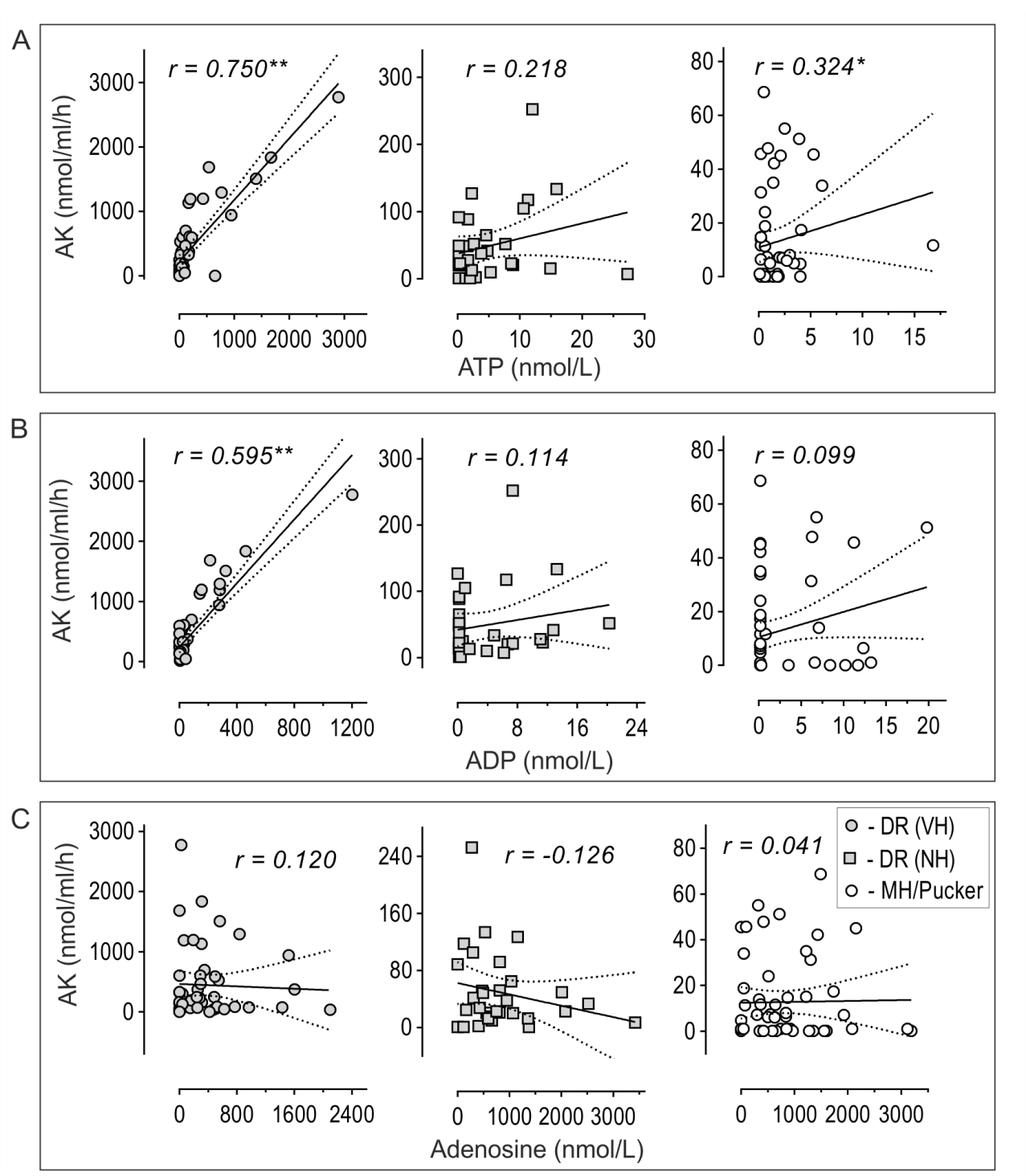
**

**Supplementary Fig. S3.** Correlation analysis between adenylate kinase (AK) activity and concentrations of purines in human vitreous. AK was assayed in VF from DR eyes with vitreous haemorrhage (VH; *closed circles*) and without haemorrhage (NH; *closed squares*) and from non-diabetic eyes operated due to MH or pucker (*open squares*). Soluble activities were further correlated with concentrations of ATP (A), ADP (B) and adenosine (C) determined in the same vitrectomized eyes. The graphs display the values determined in individual patients, best-fit linear regression lines with 95% confidence intervals, as well as Spearman’s correlation coefficients (r) computed to investigate the relationships between soluble AK and intravitreal purines. *P<0.05 and ***P* < 0.01, determined by the non-parametric two-tailed correlation analysis.


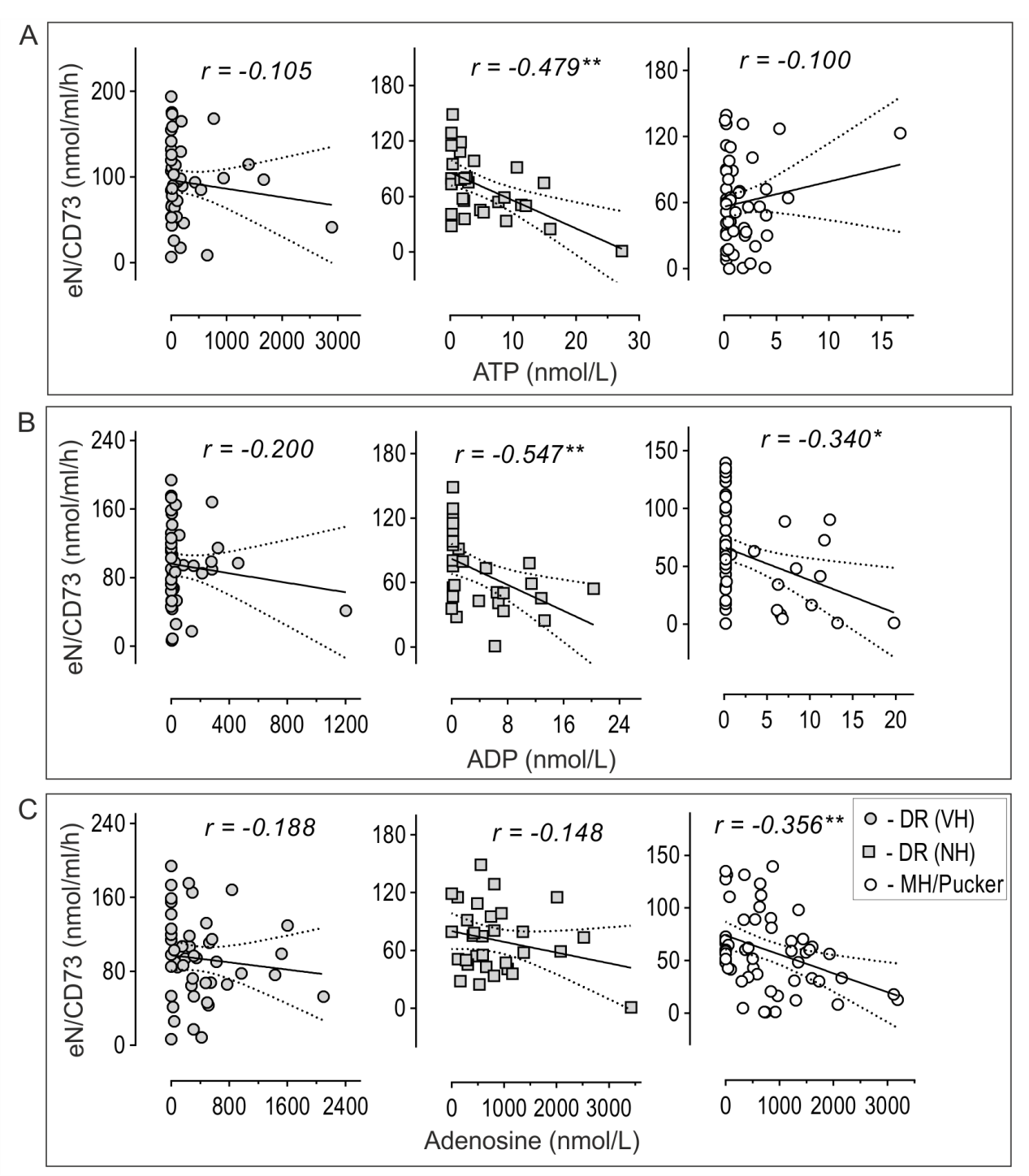


**Supplementary Fig. S4.** Correlation analysis between ecto-5’-nucleotidase/CD73 (eN/CD73) activity and concentrations of purines in human vitreous. eN/CD73 was assayed in VF from DR eyes with vitreous haemorrhage (VH; *closed circles*) and without haemorrhage (NH; *closed squares*) and from non-diabetic eyes operated due to MH or pucker (*open squares*). Soluble activities were further correlated with concentrations of ATP (A), ADP (B) and adenosine (C) determined in the same vitrectomized eyes. The graphs display best-fit linear regression lines with 95% confidence intervals, as well as Spearman’s correlation coefficients (r) computed to investigate the relationships between soluble eN/CD73 and intravitreal purines. *P<0.05 and ***P* < 0.01, determined by the non-parametric two-tailed correlation analysis.

**
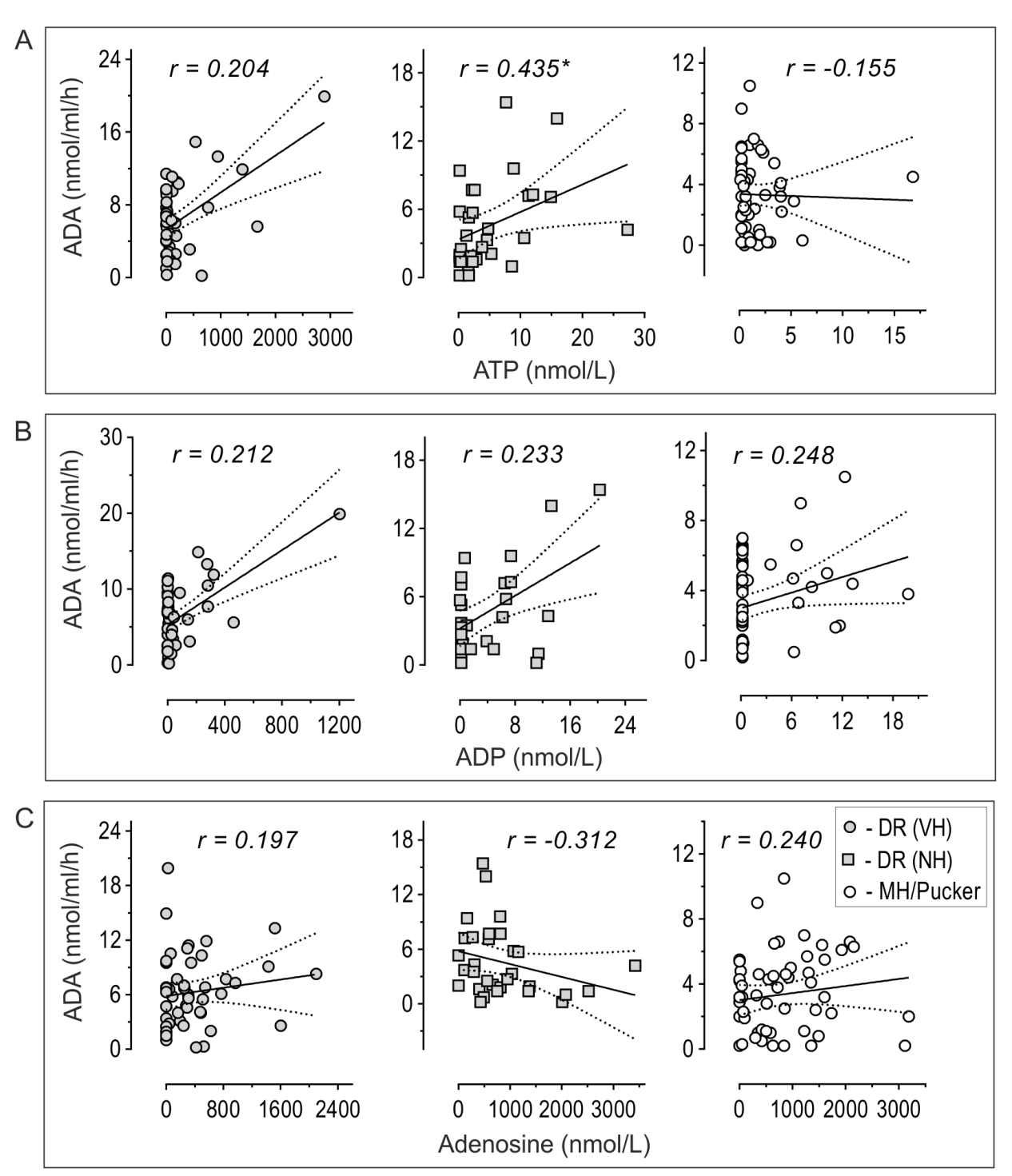
**

**Supplementary Fig. S5.** Correlation analysis between adenosine deaminase (ADA) activity and concentrations of purines in human vitreous. ADA was assayed in VF from DR eyes with vitreous haemorrhage (VH; *closed circles*) and without haemorrhage (NH; *closed squares*) and from non-diabetic eyes operated due to MH or pucker (*open squares*). Soluble activities were further correlated with concentrations of ATP (A), ADP (B) and adenosine (C) determined in the same vitrectomized eyes. The graphs display best-fit linear regression lines with 95% confidence intervals, as well as Spearman’s correlation coefficients (r) computed to investigate the relationships between soluble ADA and intravitreal purines. **P* < 0.05, determined by the non-parametric two-tailed correlation.

**
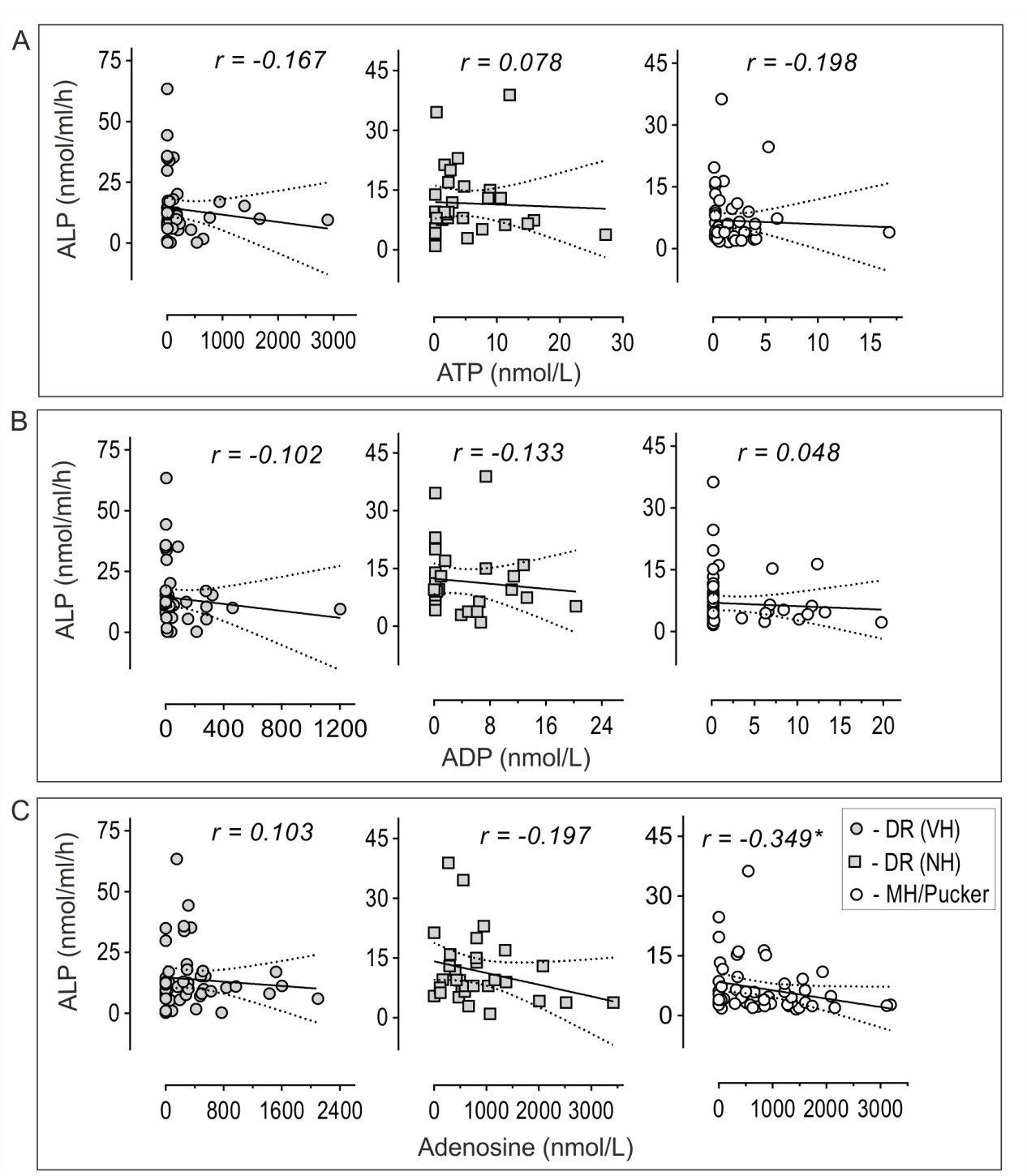
**

**Supplementary Fig. S6.** Correlation analysis between alkaline phosphatase (ALP) activity and concentrations of purines in the human vitreous. ALP was assayed in VF from DR eyes with vitreous haemorrhage (VH; *closed circles*) and without haemorrhage (NH; *closed squares*) and from non-diabetic eyes operated due to MH or pucker (*open squares*). Soluble activities were further correlated with concentrations of ATP (A), ADP (B) and adenosine (C) determined in the same vitrectomized eyes. The graphs display best-fit linear regression lines with 95% confidence intervals, as well as Spearman’s correlation coefficients (r) computed to investigate the relationships between soluble ALP and intravitreal purines. **P* < 0.05, determined by the non-parametric two-tailed correlation
